# Non-equilibrium behaviour in coacervate-based protocells under electric-field-induced excitation

**DOI:** 10.1038/ncomms10658

**Published:** 2016-02-15

**Authors:** Yudan Yin, Lin Niu, Xiaocui Zhu, Meiping Zhao, Zexin Zhang, Stephen Mann, Dehai Liang

**Affiliations:** 1 Beijing National Laboratory for Molecular Sciences, MOE Key Laboratory of Polymer Chemistry and Physics, College of Chemistry and Molecular Engineering, Peking University, Beijing 100871, China; 2 Beijing National Laboratory for Molecular Sciences, MOE Key Laboratory of Bioorganic Chemistry and Molecular Engineering, College of Chemistry and Molecular Engineering, Peking University, Beijing 100871, China; 3 Center for Soft Condensed Matter Physics and Interdisciplinary Research, Soochow University, Suzhou 215006, China; 4 Centre for Protolife Research, School of Chemistry, University of Bristol, Bristol BS8 1TS, UK

## Abstract

Although numerous strategies are now available to generate rudimentary forms of synthetic cell-like entities, minimal progress has been made in the sustained excitation of artificial protocells under non-equilibrium conditions. Here we demonstrate that the electric field energization of coacervate microdroplets comprising polylysine and short single strands of DNA generates membrane-free protocells with complex, dynamical behaviours. By confining the droplets within a microfluidic channel and applying a range of electric field strengths, we produce protocells that exhibit repetitive cycles of vacuolarization, dynamical fluctuations in size and shape, chaotic growth and fusion, spontaneous ejection and sequestration of matter, directional capture of solute molecules, and pulsed enhancement of enzyme cascade reactions. Our results highlight new opportunities for the study of non-equilibrium phenomena in synthetic protocells, provide a strategy for inducing complex behaviour in electrostatically assembled soft matter microsystems and illustrate how dynamical properties can be activated and sustained in microcompartmentalized media.

## Abstract (web summary)

Synthetic cells are designed to mimic basic life processes, but it is challenging to prepare cells that can persist and operate at non-equilibrium conditions. Here, Yin *et al.* show complex dynamical behaviors of protocells made by exposing polylysine and single-stranded DNA droplets to an electric field.

## Graphical abstract

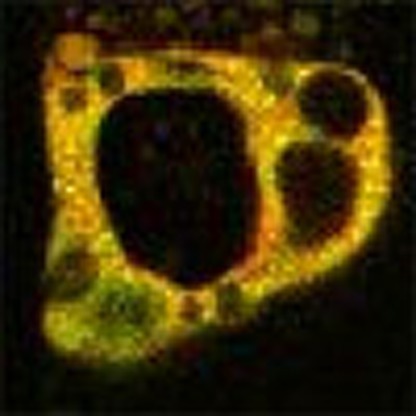

The design and construction of rudimentary forms of synthetic cell-like entities (protocells) exhibiting controlled reactivity, internal structuration and adaptive functionality are providing new approaches to protocell modelling^1^,^2^,^3^, the fabrication of integrated materials micro-ensembles^4^,^5^,^6^, and mechanisms related to prebiotic organization and the origin of life on the early Earth^7^. Functional protocells have been prepared by membrane self-assembly of amphiphilic organic molecules (polymer^8^,^9^ or lipid^10^,^11^ vesicles), surface-functionalized inorganic nanoparticles (colloidosomes)^12^,^13^ and protein–polymer nano-conjugates (proteinosomes)^14^; layer-by-layer membrane assembly of counter-charged polyelectrolytes^15^,^16^; and microphase separation of membrane-free liquid microdroplets by complex coacervation^17^. Hybrid protocells based on membrane-coated coacervate droplets with homogenous^18^ or subdivided interiors^19^,^20^, or lipid vesicles containing discrete polymer-enriched internalized domains^21^,^22^, have also been recently reported. Significantly, these strategies are almost exclusively based on the static assembly and confinement of molecular and nanoscale components that operate under close to equilibrium conditions. As a consequence, even the most highly integrated protocellular systems are functionally compromised compared with basic life processes, which occur under a continuous flux of energy and matter exchange.

Thus, a major challenge in synthetic protocell research involves the development of methodologies that enable the sustained activation of chemical micro-ensembles such that they persist and function under non-equilibrium conditions. While modularized systems can be locally excited, for example, by light-driven pumping of protons across a targeted vesicle membrane to induce gene expression^23^, energization at a non-local level requires the maintenance and coupling of chemical fuel gradients to internalized protometabolic processes, or alternatively, the indiscriminate excitation of the protocell medium by an externally applied field. In this regard, protocell models based on coacervate liquid microdroplets are distinctive for their molecularly crowded, reduced dielectric constant aqueous interiors that consist of a highly enriched matrix of electrostatically interacting counter-charged polyelectrolytes^17^,^24^. These protocell models are established under equilibrium conditions, and therefore exhibit no complex dynamical properties. However, as charged macromolecular complexes are sensitive to electrohydrodynamic forces^25^,^26^, coacervates can be globally excited by exposure to an applied electric field^27^,^28^,^29^, suggesting that it should be possible to use electric fields to sustain and control the activation of coacervate-based protocells under non-equilibrium conditions. Previous studies on the induced mobility of coacervate microdroplets prepared from high–molecular-weight (2,000 bp) double-stranded DNA have been reported^29^, although no complex internalized behaviour was observed.

Here we demonstrate that the electric-field-induced energization of spatially confined coacervate microdroplets containing a mixture of oppositely charge polypeptides and short single strands of DNA provides a step towards the design and construction of synthetic protocells capable of exhibiting a range of complex behaviours under non-equilibrium conditions. By confining the droplets within a microfluidic channel and applying a range of electric field strengths, we produce linear arrays of membrane-free protocells that undergo cycles of transient subcompartmentalization, dynamical fluctuations in size and shape, chaotic processes of growth and fusion, and unidirectional movement. We show that excitation of the protocells leads to a continuous exchange of matter with the environment via repetitive cycles of vacuole nucleation, growth and expulsion, spontaneous ejection and sequestration of microdomains of the coacervate matrix, directional capture of solute molecules, and pulsed enhancement of enzyme cascade reactions. Switching off the electric field immediately arrests the dynamical behaviour, and recapitulates the equilibrium structure and form of the protocells. Taken together, our results highlight new opportunities for the study of non-equilibrium phenomena in synthetic protocell research, provide a novel strategy for inducing complex behaviour in electrostatically assembled soft matter microsystems and illustrate how dynamical properties can be activated and sustained in microcompartmentalized media.

## Results

### Electric field excitation of coacervate-based protocells

**Figure 1:**
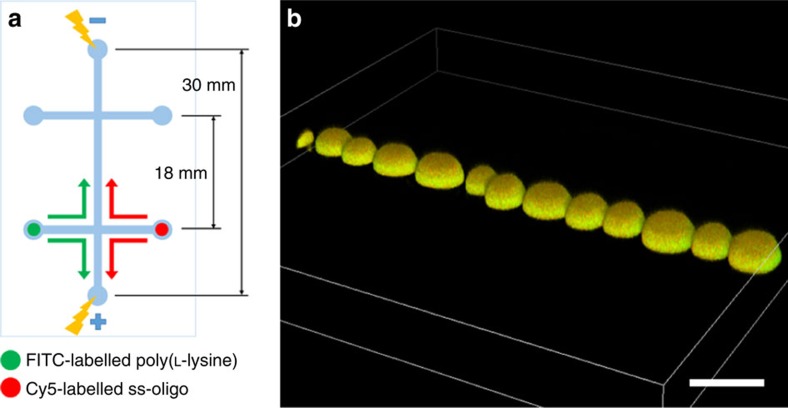
Experimental set-up. (**a**) Schematic showing microfluidic chip layout and flow of solutions. The microchannels were 80 × 25 μm in width and depth, respectively, and etched on a glass chip. FITC–PLL and Cy5–ss-oligo were placed in the opposite sample reservoirs and allowed to mix under flow at room temperature to produce an array of fluorescent PLL/ss-oligo coacervate microdroplets along the central channel. The electric field was then applied along the microfluidic channel. (**b**) 3D confocal fluorescence microscopy reconstruction showing linear array of discrete PLL/ss-oligo droplets prepared at a charge ratio of 1:1 in a microfluidic channel in the absence of an electric field. Adherence of the coacervate droplets to the channel wall due to gravity gives rise to the observed hemispherical morphology. Scale bar, 20 μm.

We prepare coacervate microdroplets within a microfluidic channel by flow-induced mixing of cationic poly-L-lysine (PLL, *M*_W_=30,000–70,000) and a negatively charged single-stranded oligodeoxynucleotide (ss-oligo, *M*_W_=6,387, 21 nt) in 0.01 M phosphate buffer, and then apply an electric field of variable strength along the microfluidic channel (Fig. 1a). The PLL and ss-oligo are fluorescently labelled with fluorescein isothiocyanate (FITC) and 1,1-*bis*(3-hydroxypropyl)-3,3,3,3-tetramethylindodicarbocyanine (Cy5), respectively, to assist imaging of the droplets within the microfluidic channel. In the absence of an electric field, room temperature mixing of the PLL and ss-oligo at a charge ratio of ≈1:1 produces a linear array of discrete coacervate microdroplets that are typically 20 μm in diameter and stable with respect to coalescence. Three-dimensional (3D) confocal fluorescence microscopy images reveal uniform fluorescence intensity throughout the droplets, indicating that the complex coacervates are structurally homogeneous in the absence of an electric field (Fig. 1b).

**Figure 2:**
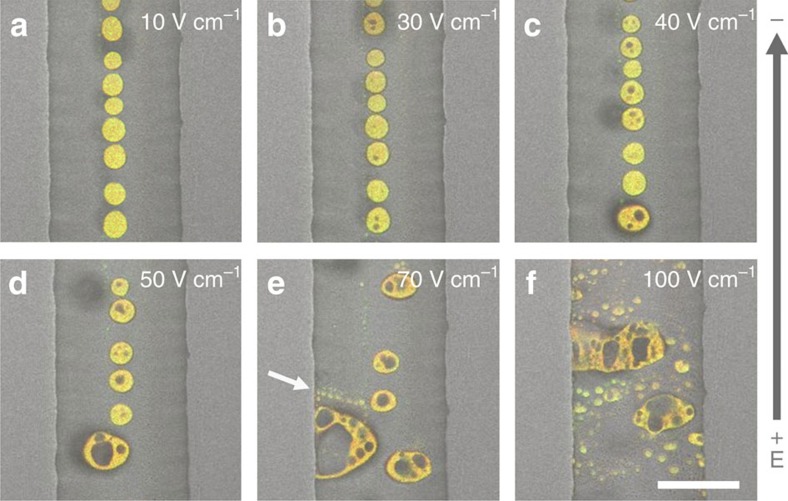
Electric field excitation of coacervate-based protocells. Video images showing PLL/ss-oligo coacervate droplets prepared in a microfluidic channel and then exposed to an applied electric field (*E*) of varying strength. (**a**) At *E*=10 V cm^−1^, showing a stable array of fluorescent coacervate droplets with homogeneous size, structure and morphology. (**b**,**c**) At *E*=30 or 40 V cm^−1^, showing immobile arrays of coacervate microdroplets containing small numbers of subcompartments (localized non-fluorescent regions). The vacuoles nucleate, grow and disappear in a cyclical process of matter exchange with the environment without significantly influencing droplet size and shape (see Supplementary Movies 1–3 for more details). (**d**,**e**) At *E*=50 or 70 V cm^−1^, showing a destabilized array of motile protocells with co-existing multiple vacuoles and increased polydispersity due to droplet fusion. Corresponding videos (Supplementary Movies 4 and 5) show rapidly fluctuating changes in the shape and size of individual droplets as they move towards the negative electrode. Arrow in **e** highlights the presence of fluorescent ejectiles that were released in the direction of the electric field from the adjacent multi-compartmentalized droplet; the ejectiles were subsequently sequestered by the same droplet at a later time interval. (**f**) At 100 V cm^−1^, showing transversely stretched multi-vacuole-containing protocells and extensive amounts of ejected coacervate matrix due to the high electrohydrodynamic forces. Scale bar, 50 μm.

Switching on the electric field (*E*) above a critical value has a remarkable influence on the structure and dynamics of the preformed PLL/ss-oligo coacervate microdroplets. While the microdroplets remain essentially unchanged at *E*<20 V cm^−1^ field, increasing the electric field to 30 or 40 V cm^−1^ results in the cyclical appearance and disappearance of non-fluorescent vacuoles within the interior of the coacervate phase (Fig. 2a–c; Supplementary Movies 1–3). Under these conditions, subcompartmentalization is repeatedly initiated at a small number (typically *n*=1–3) of randomly located sites in droplets that remain effectively unchanged in overall size and shape, and immobile within the microfluidic channel. In contrast, major fluctuations in droplet morphology and dynamics occur at *E*=50 and 70 V cm^−1^ due to an increased intensity in the growth and expulsion of the vacuoles, which are associated, respectively, with cycles of expansion and contraction of the coacervate droplets. As a consequence, the energized droplets often undergo fusion to produce larger membrane-free protocells with co-existing multiple subcompartments (Fig. 2d). The structural and morphological fluctuations are sufficiently intense that fluorescent microparticles of the coacervate medium are ejected from and then consumed by the larger droplets during contraction and swelling, respectively (Fig. 2e). Phenomenologically, the energized protocells exhibit ‘life-like' chaotic behaviour involving unidirectional movement (electric field induced), morphogenesis (rapid fluctuations in size and shape), transient subcompartmentalization, fusion and growth, and continuous exchange of matter with the environment (vacuole growth/expulsion, matrix ejection/sequestration) (Supplementary Movies 4 and 5). Furthermore, at 100 V cm^−1^, the electrohydrodynamic force is strong enough to stretch the subdivided droplets in a direction normal to the electric field (Fig. 2f; Supplementary Movie 6). Significantly, the repetitive formation of the vacuoles is stopped immediately the electric field is switched off.

### Vacuole formation and protocell dynamics

**Figure 3:**
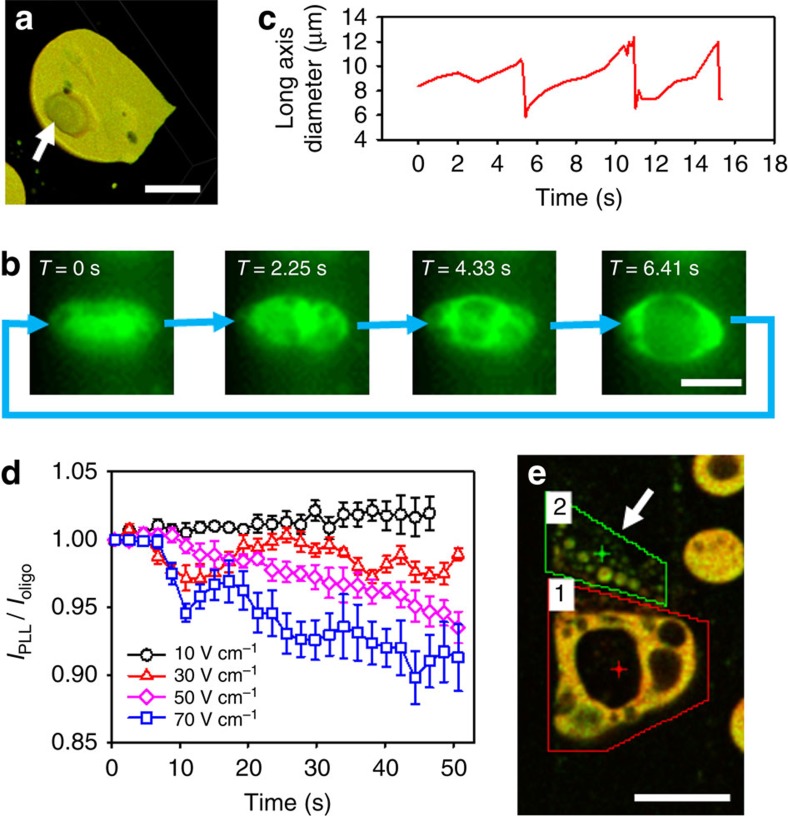
Vacuole formation and protocell dynamics in applied electric fields. (**a**) 3D confocal fluorescence microscopy reconstruction of a PLL/ss-oligo coacervate microdroplet exposed to an electric field of 70 V cm^−1^ showing the presence of a single 18-μm-sized vacuole (solid arrow). Scale bar, 20 μm. (**b**) Fluorescence microscopy images showing a time sequence of events leading to vacuole nucleation, fusion and growth within a single PLL/ss-oligo microdroplet placed in an electric field of 80 V cm^−1^. Contact of the vacuole with the droplet surface releases the subcompartment into the external medium and initiates a new cycle of matter transfer. Scale bar, 10 μm. (**c**) Plot of droplet size against time for three cycles of vacuole growth within and expulsion from an individual protocell at *E*=80 V. (**d**) Plots of relative fluorescence intensity (*I*_PLL_*/I*_ss-oligo_) against time for individual PLL/ss-oligo microdroplets exposed to electric fields ranging from 10 to 70 V cm^−1^. Values for *I*_PLL_ and *I*_oligo_ were normalized to their initial values recorded before the electric field was switched on (*t*=0). Error bar: s.e. mean. (**e**) Confocal fluorescence microscopy image showing large multi-compartmentalized PLL/ss-oligo protocell (region 1) and ejected microparticles (region 2) at 70 V cm^−1^. Fluorescence channels for FITC–PLL (green) and Cy5-tagged ss-oligo (red) are combined in the images. Analyses of the relative fluorescence intensities indicate that the concentration of PLL is highest in the expelled particles. Scale bar, 20 μm.

We use fluorescence microscopy to investigate the nature of vacuole formation within the PLL/ss-oligo protocells. 3D confocal fluorescence microscopy images confirm the presence of internal cavities in the subdivided droplets (Fig. 3a). The vacuoles nucleate randomly inside the protocells, rapidly grow in size, fuse if in the presence of more than one subcompartment and then disappear on contact with the surface of the coacervate microdroplet (Fig. 3b). Release of the vacuole contents into the bulk aqueous phase is followed by a further cycle of nucleation, growth and ejection of the subcompartments, which continue without loss of droplet integrity until the electric field is switched off. The lifetime associated with vacuole growth and expulsion is determined within individual droplets from the video recordings. As the fluorescence intensity associated with the vacuoles is close to the background level, fluorescence line scans across subdivided droplets give a series of peaks corresponding to the edges of the coacervate-rich areas (Supplementary Fig. 1). The size of the vacuoles is determined by measuring the distance between the fluorescence intensity peaks at various time intervals. As shown in Fig. 3c, the vacuole lifetime is of the order of around 5 s in an electric field of 80 V cm^−1^.

Changes in the composition of the coacervate matrix during repeated cycles of swelling and contraction accompanying vacuole growth and expulsion at electric fields of 10–70 V cm^−1^ are investigated by fluorescence microscopy measurements on individual droplets. Time-dependent measurements of the relative fluorescence intensity, *I*_r_=*I*_PLL_/*I*_oligo_, are used as an approximate index of how the PLL/ss-oligo ratio in the droplets changes over a period of 50 s (Fig. 3d). No significant change in the normalized ratio is observed at 10 V cm^−1^, indicating that the coacervate matrix is compositional stable in the vacuole-free protocells. In contrast, onset of subcompartmentalization in droplets at *E*>30 V cm^−1^ results in a time-dependent reduction in *I*_r_ that fluctuates probably due to matter exchange with the external environment during the swelling/contraction cycles, and becomes more pronounced as the electric field is increased. For example, a 10% drop in the value of *I*_r_ is observed after 50 s at 70 V cm^−1^, indicating that a considerable amount of PLL is released as the protocells undergo rapid fluctuations in size and shape. To determine when and how the PLL is released, we measure the *I*_r_ values associated with the fluorescent microparticles produced *in situ* by ejection from a neighbouring parent droplet at 70 V cm^−1^ (Fig. 3e). The average *I*_r_ value for the ejected particles (region 2 in Fig. 3e and Supplementary Fig. 2) is ∼40% higher than that of the parent droplet (region 1 in Fig. 3e), indicating that PLL is preferentially released from the coacervate matrix during repeated cycles of vacuolization. As the PLL chain length is considerably greater than that for the ss-oligo, uncomplexed loops of PLL within the coacervate matrix will be subjected to higher dragging forces, and therefore preferentially expelled during the excessive fluctuations in droplet morphology observed at relatively high electric fields.

### Molecular uptake during vacuolarization

**Figure 4:**
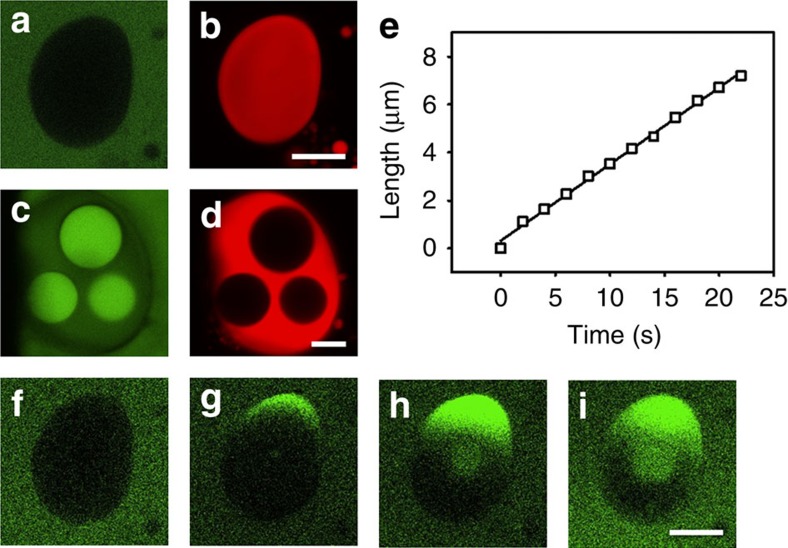
Molecular uptake during vacuolarization. (**a**–**d**) Confocal fluorescence microscopy images showing a single PLL/Cy5-labelled ss-oligo coacervate microdroplet prepared in a microfluidic channel with addition of the water-soluble, green fluorescent dye, calcein (20 μM) before (**a**,**b**) and after (**c**,**d**) activation in an applied electric field at 30 V cm^−1^. No uptake of calcein is observed in the microdroplet before excitation (**a**), which shows only homogeneous red fluorescence associated with the Cy5–ssDNA component (**b**). In contrast, calcein is observed in the vacuoles but not in the coacervate matrix when the droplet is exposed to an electric field (**c**,**d**), indicating that the vacuoles are formed by water ingress from the external environment. (**e**–**i**) Time-dependent uptake of calcein in the microdroplets under energization after 1 (**f**), 15 (**g**), 30 (**h**) and 45 s (**i**). Positions of the calcein wave front (**e**) were used to calculate dye mobility in the droplets. Scale bar, 10 μm.

Other studies indicate that not only are components of the droplet selectively expelled but also solute molecules in the external medium can be taken up directionally and accumulated inside the droplets in the presence of an electric field. Using a water-soluble fluorescent dye (calcein) as a model molecule, we monitor the migration behaviour and spatial distribution of the solute during the vacuolarization process. Negligible uptake of calcein into the coacervate matrix of the microdroplets is observed by confocal fluorescence microscopy in the absence of an electric field (Fig. 4a,b), consistent with the relative hydrophobic nature of the PLL/ss-oligo phase. In contrast, calcein is observed in the vacuoles but not in the coacervate matrix when the droplet is exposed to an electric field (Fig. 4c,d). As the vacuolization process is cyclical, transportation of the external aqueous phase into and out of the droplet occurs as long as the electric field is switched on. Moreover, once the electric field is applied, calcein molecules migrate into the coacervate phase from one end of the droplet along the direction of electric field (Fig. 4e–i; Supplementary Movie 7). A linear time-dependent relationship is observed for the advancing wave front of dye molecules, indicating an almost constant velocity for calcein uptake (Fig. 4e). The calculated mobility within the droplets (−3.2 × 10^−10^ m^2^ s^−1^ V^−1^) is significantly lower than the value observed in free solution (−6.0 × 10^−8^ m^2^ s^−1^ V^−1^)^30^. As a consequence, the concentration of calcein in the droplet as indicated by the fluorescence intensity is considerably higher than that in the surrounding phase.

Transport of the external aqueous phase during vacuolization is driven by osmotic pressure generated by an elevated local ion concentration in the droplets exposed to an electric field. Droplets prepared at PLL and ss-oligo at higher concentrations (PLL: 4.0 mg ml^−1^, ss-oligo: 6.0 mg ml^−1^) contain domains rich in ions due to incomplete complexation^31^. Indeed, under these conditions, vacuolization is observed in the absence of an electric field when the continuous phase is replaced by a buffer solution (Supplementary Fig. 3). The vacuoles are mainly located in the PLL-enriched domain of the forming droplets due to the longer chain length of the cationic polymer, which gives rise to an increased number of unneutralized loops^31^, and higher ionic concentration. Significantly, enhanced dissociation of the polyelectrolyte complex occurs in the presence of an electric field, which in turn increases the local concentration of free ions within the droplets, enabling vacuolization in the droplet at lower polymer concentrations. As the imbibed water does not mix with the viscoelastic coacervate phase, the vacuole grows under a compressive force, but because there is no restraining membrane at the droplet surface is stochastically released back into the continuous phase, contracting the droplet and initiating the next cycle of matter transfer. The presence of elevated salt concentrations in the continuous phase or substitution of the ss-oligo for a more strongly bonded double-stranded oligo-DNA counterpart suppresses vacuole formation (data not shown) by offsetting the osmotic pressure or curtailing disassociation of the complex, respectively.

### Electric-field-mediated enhancement of enzyme reactions

**Figure 5:**
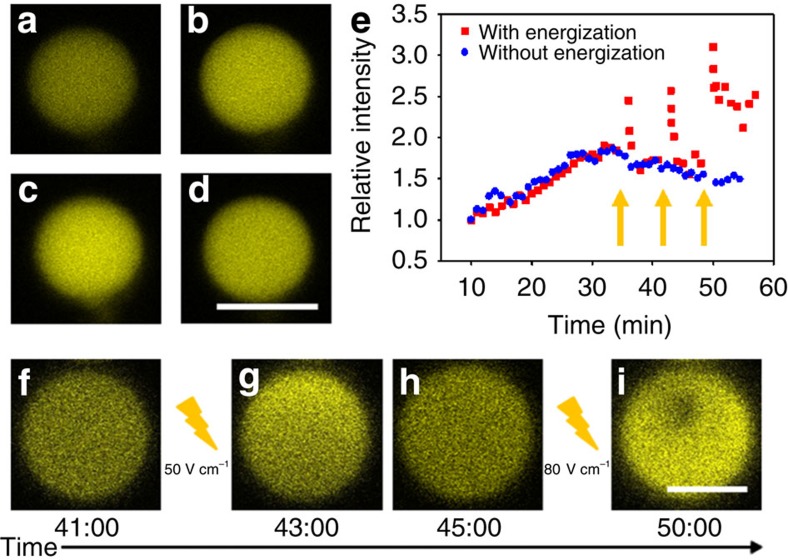
Electric-field-mediated enhancement of an enzyme cascade reaction. (**a**–**d**) Confocal fluorescence microscopy images of a single PLL/ss-oligo coacervate microdroplet containing GOx/HRP enzymes and showing 2,3-DAP fluorescence in the absence of an electric field 10 (**a**), 20 (**b**), 30 (**c**) and 50 min (**d**) after addition of *o*PD and β-D-glucose substrates; scale bar, 10 μm. (**e**) Relative intensities of 2,3-DAP produced in droplets subjected to intermittent energization (red squares) or without energization (blue dots). An electric field of 50 V cm^−1^ was applied at 35 and 42 min for a period of 1 min, and at 49 min using a field of 80 V cm^−1^ for 1 min (arrows). (**f**–**i**) Confocal fluorescence microscopy images of 2,3-DAP fluorescence intensity just before energization (**f**), immediately after energization in an electric field of 50 V cm^−1^ showing increase in fluorescence intensity and redistribution of 2,3-DAP (upper region of the droplet) (**g**), 2 min after the 50 V cm^−1^ electric field is switched off showing decrease in fluorescence intensity (**h**), and immediately after energization in an electric field of 80 V cm^−1^ showing high-intensity 2,3-DAP fluorescence in the coacervate matrix but not in the vacuole (**i**). Scale bar, 5 μm.

Inspired by the directional movement, accumulation and throughput of molecules observed for microdroplets under electric field energization, we explore the possibility of undertaking enzyme-mediated chemical transformations^19^ in the activated medium. Glucose oxidase (GOx) and horseradish peroxidase (HRP) are encapsulated in the coacervate droplets during preparation, and a mixture of *o*-phenylenediamine (*o*PD, substrate for HRP) and β-D-glucose (substrate for GOx) is injected into the microfluidic channel to replace the original buffer. Diffusion of oPD and β-D-glucose into the coacervate droplets initiates the enzyme-mediated tandem reaction to produce a fluorescent final product, 2,3-diaminophenazine (2,3-DAP) that is monitored in individual droplets over time (Supplementary Table 1). A steady increase in the 2,3-DAP fluorescence intensity followed by a slow decrease after 35 min is observed for enzyme-containing droplets in the absence of an electric field (Fig. 5a–d; Fig. 5e (blue curve)). We attribute the fall off in intensity to the formation of a quasi-steady state between 2,3-DAP production within the droplet and release of the product to the external environment. This behaviour is not observed for cascade reactions undertaken in bulk solution; in this case, a continuous increase in intensity is observed over a time period of 60 min. Significantly, a sharp increase in 2,3-DAP fluorescence intensity in the droplets is observed when an electric field of 50 V cm^−1^ is applied for 1 min (Fig. 5e (red curve); Fig. 5f–i). The electric field also redistributes the positively charged 2,3-DAP molecules towards one region of the droplet (Fig. 5g; Supplementary Fig. 4), and the fluorescence intensity decreases to a steady state value within a few minutes after the electric field (50 V cm^−1^) is switched off (Fig. 5e), such that the enzyme reaction can be pulsed by intermittent switching on and off of the electric field (Fig. 5e). Control experiments undertaken in the absence of the enzymes show negligible changes in fluorescence, indicating that the observed increase in fluorescence intensity originates specifically from the formation of 2,3-DAP (Supplementary Fig. 5).

We attribute the electric-field-mediated enhancement of the enzyme cascade reaction rate to the triggered rapid uptake and accumulation of *o*PD and β-D-glucose within the droplets due to electric-field-induced vacuolization. Interestingly, at a higher voltage of 80 V cm^−1^, where dynamic fluctuations in the size and shape of the droplet occur, the overall instantaneous intensity of 2,3-DAP is considerably higher than at 50 V cm^−1^ after the droplets are energized for 1 min. Moreover, the 2,3-DAP product is mainly distributed outside the substrate-containing aqueous vacuole and within the enzyme-containing coacervate matrix (Fig. 5f–i). Overall, these experiments demonstrate that electric field energization of the microdroplets can be used to trigger immediate rate enhancements in spatially confined enzyme reactions, and that vacuolization offers a transient means to partially separate reactants and product molecules associated with the biocatalytic cascade.

## Discussion

Our results highlight a novel experimental protocol for the controlled electric field excitation of coacervate-based microdroplets that offers new opportunities for the study of non-equilibrium phenomena in synthetic protocells. The ability to study protocells under sustained energization is in marked contrast to current models that are limited by their design and function under close to equilibrium conditions. Our studies therefore offer a step towards a better representation of minimal life processes operating under a continuous flux of energy and exchange of matter with the environment. In particular, the possibility of controlling the onset, duration and termination of complex dynamical behaviour such as morphogenesis, transient subcompartmentalization (vacuolization), growth and fusion, spontaneous ejection and sequestration of matter, directional capture of solute molecules, and pulsed enhancement of enzyme cascades should provide an interesting context in which to study chemical reactivity and signalling in protocell communities. For example, coacervate microdroplets exhibit key biomimetic characteristics such as high levels of chemical enrichment^17^,^24^, uptake and retention of enzymes and DNA without droplet transfer or exchange^32^,^33^, increased rates of enzymatic transformations^17^,^24^,^34^ and surface properties compatible with facilitating membrane assembly^18^,^19^,^20^. The exploitation of these properties in energized protocells should provide a route towards increased complexity and functionality that could have implications for new developments in activated microstorage and delivery, and non-equilibrium microreactor technologies.

The demonstration of diverse non-equilibrium phenomena in coacervate microdroplets exposed to an electric field offers a step towards novel examples of reactive soft matter. Electrohydrodynamic forces within the molecularly crowded interior of the protocells facilitate disassociation the PLL/ss-oligo complex to produce a charge-activated matrix and osmotic gradient at the droplet surface that drive a cyclical exchange of matter with the environment. Our results suggest that loosely associated polyelectrolytes are particularly sensitive to the electric field, and that the composition and structural heterogeneity of the coacervate matrix are important criteria in determining their non-equilibrium behaviour. As coacervate microdroplets can be prepared across a range of polyelectrolyte charge ratios^24^, and kinetically trapped structural states^29^,^35^, changes in experimental methodology (non-stoichiometric mixtures, ageing times) need to be considered in detail when investigating their dynamical properties.

Finally, we speculate that our results could be of interest to areas of origin of life research, particularly in stimulating new ideas concerning the activation and subdivision of prebiotic assemblies on the early Earth. The spontaneous microphase separation and chemical enrichment in water of counter-charged biologically relevant molecules such as ATP, DNA, RNA and polypeptides^17^,^18^,^33^ to produce coacervate droplets is consistent with a possible mechanism of membrane-free prebiotic compartmentalization^17^,^36^. Condensates of these charged components are prevalent in extant organisms^37^,^38^,^39^, and electric fields exist both extracellularly and intracellularly^40^,^41^, and play a role in dynamical processes such as tissue morphogenesis and regeneration^42^. However, although electric discharges (lightning) have been considered a plausible mechanism for the synthesis of certain prebiotic molecules^43^,^44^, the possibility that electric fields generated in the ground from earthquake activity^45^, or streaming potentials in porous rocks^46^ could facilitate the energization of early cells, or influence their dynamical behaviour and trafficking of matter remains unknown.

## Methods

### Experimental set-up

A ss-oligo (*M*_W_=6,387) with a 21 nt random sequence and a Cy5 fluorescent tag linked at the 5′-terminus was purchased from Invitrogen Inc. FITC-labelled PLL (*M*_W_=30,000–70,000) was purchased from Sigma-Aldrich (St Louis, MI, USA) and used as received. Microchannels with a double-cross layout were etched on a glass chips by soft lithography. The ss-oligo (1.5 mg ml^−1^) and PLL (1.0 mg ml^−1^) were dissolved in phosphate buffer (0.01 M, pH 6.86) and loaded into the opposite wells of a microfluidic channel device (channel dimensions; 80-μm width × 25-μm depth) that was previously washed in sequence with NaCl (1 M), NaOH (1 M), HCl (1 M), deionized water and 1% (w/w) phosphate-buffered polyvinylpyrrolidone (*M*_W_=30,000; 0.01 M KH_2_PO_4_–Na_2_HPO_4_, pH 6.86, 2 h) to minimize electro-osmosis flow and to prevent adsorption of analytes. The flow of the solution was driven by gravity. At the same height, the flow rates of ss-oligo and PLL were similar. They were thus mixed in the central channel of the chip with equal volume. Discrete coacervate microdroplets with 1:1 charge ratio were formed by mixing ss-oligo and PLL at 1.5 and 1.0 mg ml^−1^, respectively. (Varying the charge ratio or aging time after mixing of the polyelectrolytes was used to change the mean droplet diameter if required.) After 3 min, residual aqueous oligoDNA and PLL were replaced in the channel by phosphate buffer. An electric field, typically between 10 and 200 V cm^−1^, was then applied along the microfluidic channel by using a ZDMCI6-1 Microfluidic Chip Detection System (Hang-zhou Syltech Technology Co., Ltd). The changes in droplet morphology, organization and dynamics were recorded by using an Inverted Fluorescence Microscope (IX71, Olympus, USA) with a U-MWIBA module (excitation filter BP460–495, emission filter BA510–550) equipped with an EMCCD (Evolve512, Photometric, USA), or a Laser Scanning Confocal Microscope (LSCM, A1R-si, Nikon, Japan) for video imaging or 3D image reconstruction, respectively. The excitation and emission wavelengths were 638 and 700 nm (ss-oligo), and 488 and 515 nm (PLL), respectively.

Similar experiments were undertaken using coacervate droplets prepared from a mixture of PLL and a double-stranded oligodeoxynucleotide. The latter was prepared by mixing two ss-oligos with complementary sequences (5′-CTTACGCTGAGTACTTCGATT-3′ and 5′-AATCGAAGTACTCAGCGTAAG-3′; Invitrogen Inc) at equal molar ratio, followed by heating at 95 °C for 5 min and slow cooling to room temperature.

### Monitoring of dynamical behaviour

Cyclical changes in the size of the internal compartments produced within individual coacervate microdroplets exposed to an electric field of 80 V cm^−1^ were determined by time-dependent video measurements of the distance between the two inner edges of selected vacuoles. As the fluorescence intensity associated with the vacuoles was close to the background level, fluorescence line scans across subdivided droplets gave a series of peaks corresponding to the edges of the coacervate-rich areas. The size of the vacuoles was determined by measuring the distance between the fluorescence intensity peaks at various time intervals.

Experiments to confirm the aqueous content of the vacuoles formed by the excitation of electric field were carried out with PLL/ss-oligo (Cy5 labelled) coacervate microdroplets produced as described above but in the presence of 20 μM calcein. Uptake experiments were undertaken using a similar procedure.

Time-dependent changes in the macromolecular composition of individual coacervate microdroplets exposed to electric fields ranging from 10 to 70 V cm^−1^ were monitored by determining the relative fluorescence intensity, *I*_r_=*I*_PLL_/*I*_oligo_ as an index of the PLL/ss-oligo ratio, where *I*_PLL_ and *I*_oligo_ were the fluorescence intensities of FITC-labelled PLL and Cy5-labelled DNA, respectively. Both intensities were normalized to their initial values recorded before the electric field was switched on (*t*=0). Changes in fluorescence for more than five droplets were determined, and the average values used for comparison.

### Enzyme cascade experiments

Experiments to study the influence of electric field energization of the coacervate microdroplets on spatially confined enzyme-mediated reactions were undertaken as follows. Coacervate droplets co-mixed with GOx (GOx from *Aspergillus niger*) and HRP (FITC-labelled GOx or rhodamine isothiocyanate (RITC)-labelled HRP were used in some experiments) were prepared from PLL (1.0 mg ml^−1^) and a mixture of ss-oligo (1.5 mg ml^−1^), GOx (20 μg ml^−1^) and HRP (10 μg ml^−1^). A double-component mixture of *o*PD (1 mM) and β-D-glucose (2 mM) (substrates for HRP and GOx) in 20 mM MES buffer (pH 6) was injected into the channel replacing the buffer and initiating the enzyme-mediated tandem reaction in the coacervate droplets. The intensity of the fluorescent product 2,3-DAP was measured using laser scanning confocal microscope (405 nm excitation and 530 (±30) nm emission). Unlabelled GOx or HRP was used in some experiments to avoid fluorescence resonance energy transfer.

## Additional information

**How to cite this article:** Yin, Y. *et al.* Non-equilibrium behaviour in coacervate-based protocells under electric-field-induced excitation. *Nat. Commun.* 7:10658 doi: 10.1038/ncomms10658 (2016).

## Supplementary Material

Supplementary Information Supplementary Figures 1-5 and Supplementary Table 1

Supplementary Movie 1 Confocal fluorescence microscopy video of a linear array of fluorescent PLL/ss-oligo coacervate droplets confined within a microfluidic channel and exposed to an electric field of 10 V cm^−1^. Optical and fluorescent channels were combined. Movie is shown at 5 times of real time speed at 5 frames per second. Total duration of recording was 20 seconds in real time.

Supplementary Movie 2 Confocal fluorescence microscopy video of a linear array of fluorescent PLL/ss-oligo coacervate droplets confined within a microfluidic channel and exposed to an electric field of 30 V cm^−1^. Optical and fluorescent channels were combined. Movie is shown at 5 times of real time speed at 5 frames per second. Total duration of recording was 65 seconds in real time.

Supplementary Movie 3 Confocal fluorescence microscopy video of a linear array of fluorescent PLL/ss-oligo coacervate droplets confined within a microfluidic channel and exposed to an electric field of 40 V cm^−1^. Optical and fluorescent channels were combined. Movie is shown at 5 times of real time speed at 5 frames per second. Total duration of recording was 75 seconds in real time.

Supplementary Movie 4 Confocal fluorescence microscopy video of a linear array of fluorescent PLL/ss-oligo coacervate droplets confined within a microfluidic channel and exposed to an electric field of 50 V cm^−1^. Optical and fluorescent channels were combined. Movie is shown at 5 times of real time speed at 5 frames per second. Total duration of recording was 65 seconds in real time.

Supplementary Movie 5 Confocal fluorescence microscopy video of fluorescent PLL/ss-oligo coacervate droplets confined within a microfluidic channel and exposed to an electric field of 70 V cm^−1^. Optical and fluorescent channels were combined. Movie is shown at 5 times of real time speed at 5 frames per second. Total duration of recording was 45 seconds in real time.

Supplementary Movie 6 Confocal fluorescence microscopy video of fluorescent PLL/ss-oligo coacervate droplets confined within a microfluidic channel and exposed to an electric field of 100 V cm^−1^. Optical and fluorescent channels were combined. Movie is shown at 5 times of real time speed at 5 frames per second. Total duration of recording was 50 seconds in real time.

Supplementary Movie 7 Confocal fluorescence microscopy video of fluorescent calcein molecules migrated into the coacervate phase from one end of the droplet along the direction of electric field. Movie is shown at 10 times of real time speed at 5 frames per second. Total duration of recording was 94 seconds in real time.
