# Supplementary material for: Non-equilibrium behaviour in coacervate-based protocells under electric-field-induced excitation
Source: Nat Commun. 2016 Feb 15;7:10658. doi: 10.1038/ncomms10658 (PMC4756681; doi:10.1038/ncomms10658)
Supplement: Supplementary Information — Supplementary Figures 1-5 and Supplementary Table 1 [file ncomms10658-s1.pdf]

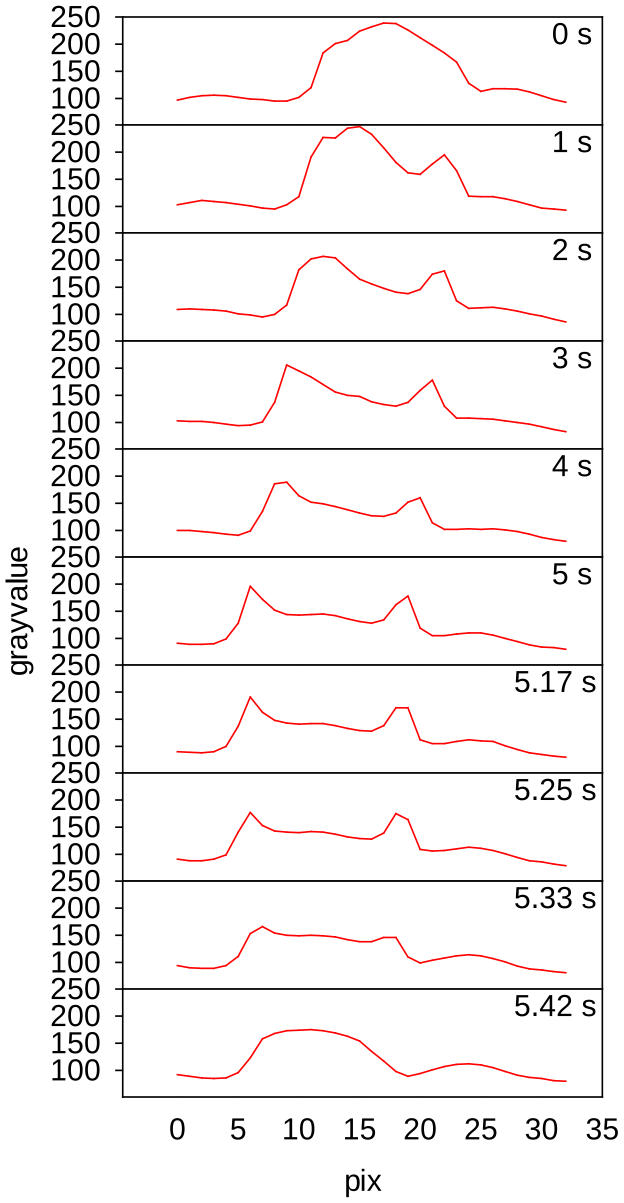

Supplementary Figure 1. Plots showing time-dependent changes in the fluorescence line scanning profiles recorded across individual droplets under going sub-division at 80 V cm^{-1}. The vacuole size was determined from the distance between the peaks. A single peak indicates that no vacuole is present at that time point.

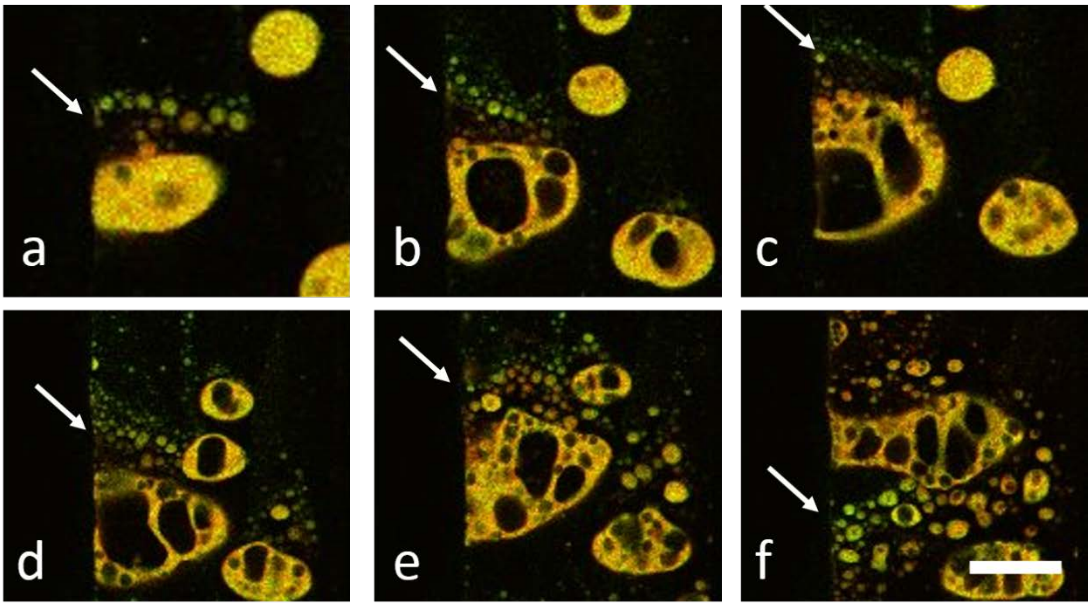

Supplementary Figure 2. Confocal fluorescence microscopy images showing preferential ejection of an array of PLL micro-particles (white arrows) from coacervate droplets under various electric fields. Fluorescence channels for FITC-PLL and Cy5-tagged ss-oligo are green and red, respectively. (**a**) image recorded at 50 V cm^{-1}; (**b**,**c**) consecutive time-dependent images at 70 V cm^{-1}, and (**d**-**f**) time sequence of droplets imaged at 100 V cm^{-1}. Scale bar = 20 µm.

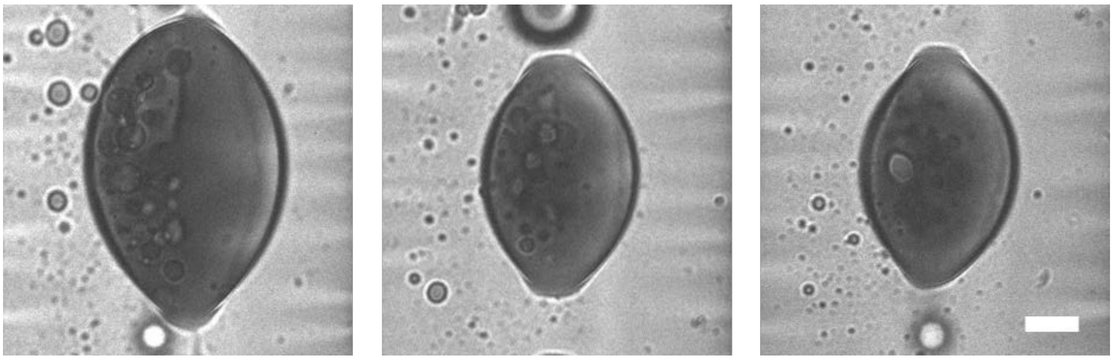

Supplementary Figure 3. Optical microscope images showing vacuolization of three different droplets driven by osmosis pressure in the absence of an electric field. The coacervate droplets were prepared at high concentrations of PLL and ss-oligo (4.0 mg mL^{-1} and 6.0 mg mL^{-1} respectively). The vacuoles are mainly located in the PLL-rich side (left) of the droplet that is produced due to directional fluid flow during the formation of droplets. Scale bar = 10 µm.

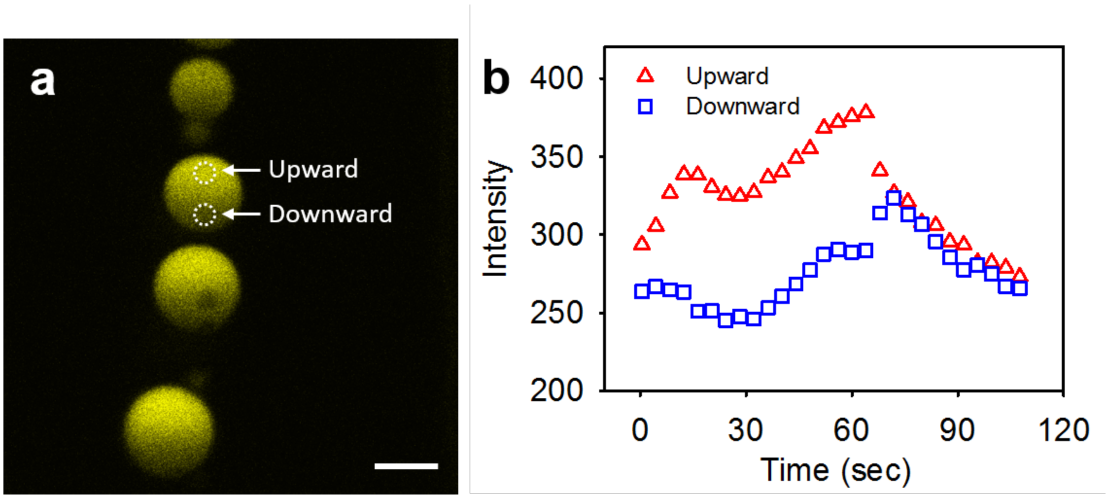

Supplementary Figure 4. (**a**) Confocal fluorescence microscopy image of PLL/ss-oligo coacervate micro-droplets containing GOx/HRP enzymes and showing 2,3-DAP fluorescence distribution during the excitation of the electric field (50 V cm^{-1}). The electric field redistributed the positively charged 2,3-DAP molecules towards one region of the droplet (the difference of fluorescence intensities between the upward and the downward part of an individual droplet). Scale bar = 10 µm. (**b**) Fluorescence intensity measured from the different regions (upward/downward) of enzyme-containing droplets during energization at 50 V cm^{-1} for 1min. The electric field was applied at time zero, and switched off at 60s. In the presence of the electric field, the upward region had a higher intensity (concentration of 2,3-DAP molecules) than the downward region due to migration of 2,3-DAP cations. The intensities become homogeneous approximately 20 s after the electric field is switched off.

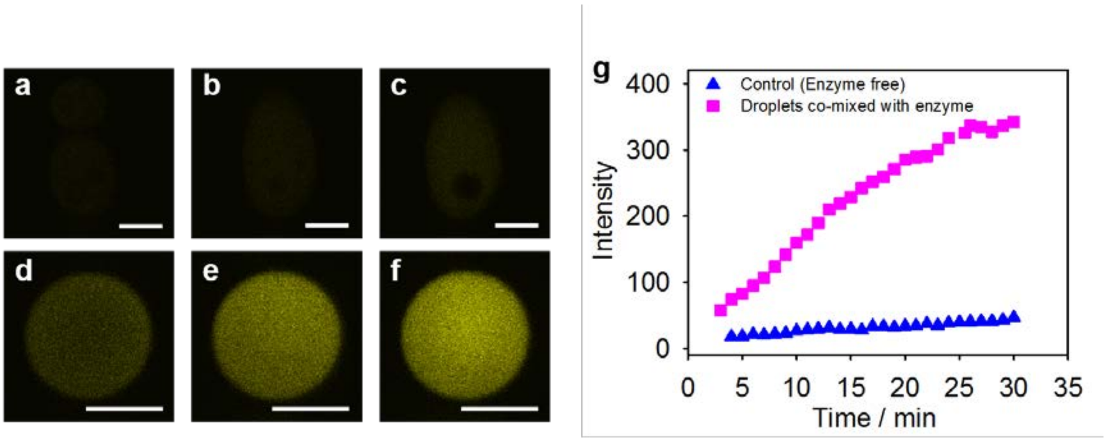

Supplementary Figure 5. (**a**-**f**)Confocal fluorescence microscopy images of PLL/ss-oligo coacervate micro-droplets without enzymes (**a**-**c**) and co-mixed with HRP and GOx (**d**-**f**). Only very weak fluorescence emission was observed at 530 (±30) nm in the absence of an electric field in 5 (**a**), 15 (**b**), and 30 (**c**) minutes after the addition of *o*PD and β-D-glucose substrates. The very weak fluorescence signal comes from the oxidization of *o*PD. The intensity is negligible compared to that from 2,3-DAP in the presence of enzymes in 5 (**d**), 15 (**e**), and 30 (**f**) minutes after the addition of substrates. Scale bar = 10 μm. (**g**)Plot showing the mean fluorescence intensity of PLL/ss-oligo coacervate micro-droplets without enzymes (triangle) and co-mixed with HRP and GOx (square). All the images and data were obtained with the same laser strength, exposure time, and Look Up Tables (LUTs) to ensure the comparability.

Supplementary Table 1. Enzyme-mediated tandem reaction and the related compounds.

Enzyme-mediated tandem reaction	D-Glucose + H_2O+O_2 $\xrightarrow{\text{GOx}}$ D-Gluconic Acid + H_2O_2<br>H_2O_2 + *o*-Phenylenediamine $\xrightarrow{\text{HRP}}$ 2,3-Diaminophenazine (Orange)
D-Glucose	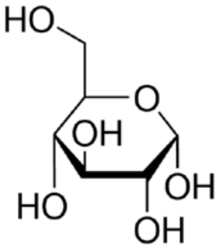
o-Phenylenediamine	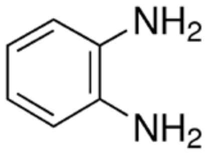
2,3-Diaminophenazine	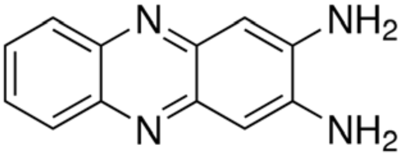
